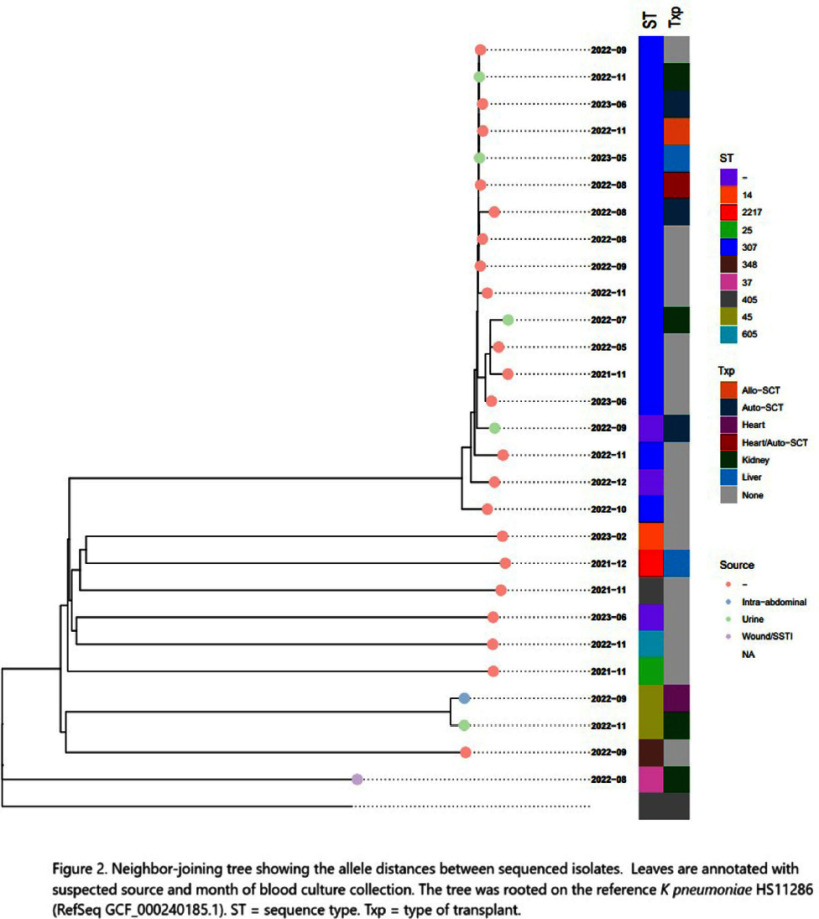# Genomic Characterization of Extended-Spectrum Beta Lactamase (ESBL)-Producing Klebsiella pneumoniae in Transplant Recipients

**DOI:** 10.1017/ash.2025.362

**Published:** 2025-09-24

**Authors:** Christina Vojtek, Alison Benton, Matthew Rodgers, David Gaston, Milner Staub, Romney Humphries, Tom Talbot, Kevin Dee, Augusto Dulanto Chiang

**Affiliations:** 1 Vanderbilt University Medical Center

## Abstract

**Background:** Multidrug-resistant organisms (MDRO), including those producing extended-spectrum beta lactamases (ESBL) are increasing. Infections, especially with MDRO, lead to increased healthcare costs. Bloodstream infections (BSI) caused by ESBL-producing Klebsiella pneumoniae increased at our institution in 2021-2022. An assessment as to whether these organisms were acquired during hospitalization or other healthcare exposures was conducted. **Methods:** The rates of ESBL-producing Klebsiella BSI per 1000 hospitalizations in 2021 and 2022 in transplant recipients and non-transplant patients were compared. From 1/1/2021 to 06/30/2023, 49 adult patients at an academic medical center had Klebsiella pneumoniae BSI with ceftriaxone resistance and a high probability of carrying an ESBL. Of these, 20 were transplant recipients and 29 were non-transplant patients. We performed whole-genome sequencing on the 28 available unique patient isolates (12 transplant; 16 non-transplant) to assess relatedness. Additional data were collected by chart review on transplant recipients. BugSeq bioinformatics pipeline and refMLST were utilized on the sequenced isolates to assess clonality, defined as ≤20 allele difference between two isolates. **Results:** The rate of ESBL-producing Klebsiella BSI increased universally from 2021 to 2022 but impacted the transplant cohort (0.3 to 3.6 per 1000 hospitalizations) more than the non-transplant cohort (0.2 to 0.5 per 1000 hospitalizations). Kidney and liver transplants were most often involved (5 out of 49 patients each). In the transplant cohort, bacteremia alone (45%, 9 out of 20) and urinary source (35%, 7 out of 20) were the most frequently identified etiologies. The most common sequence type was ST-307, accounting for 64% of total sequenced isolates (67% of transplant; 63% of non-transplant). The most common ESBL gene identified was blaCTX-M-15, identified in 24 isolates (86%). Less common resistance genes included blaSHV-12 (N=3) and blaCTX-M-3 (N=1). While there were 5 isolates within 20 allele differences (3 transplant; 2 non-transplant), they were separated in time and did not have obvious epidemiologic connections. While there was a change in TMP-SMX prophylaxis protocol during this time in kidney transplant recipients, it did not explain the increase observed in other transplant groups. **Conclusions:** There was a sharp increase in the number of BSI caused by ESBL-producing Klebsiella pneumoniae in the transplant population between 2021 and 2022. A molecular epidemiologic analysis ruled out clonal transmission from breakdown of infection prevention practices as the cause. No other common epidemiologic link was identified. This demonstrates the application of whole-genome sequencing in excluding a clonal outbreak from a common source within an institution.

**Figure:**
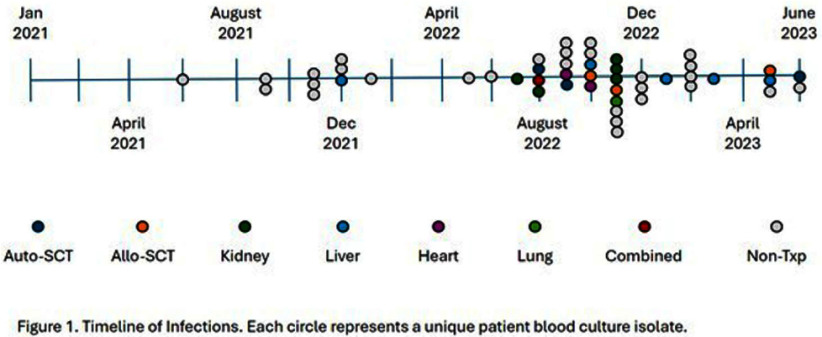

**Figure:**